# Crystal Structure, Infrared Spectrum and Elastic Anomalies in Tuperssuatsiaite

**DOI:** 10.1038/s41598-020-64481-8

**Published:** 2020-05-05

**Authors:** Francisco Colmenero, Jiří Sejkora, Jakub Plášil

**Affiliations:** 1 Instituto de Estructura de la Materia (IEM-CSIC), C/ Serrano 123, 28006 Madrid, Spain; 2 Mineralogicko-petrologické oddělení, Národní museum, Cirkusová 1740, 193 00, Praha 9, Czech Republic; 3 Institute of Physics ASCR, v.v.i., Na Slovance 2, 182 21, Praha 8, Czech Republic

**Keywords:** Theory and computation, Condensed-matter physics, Structure of solids and liquids, Materials science, Mechanical properties

## Abstract

The full crystal structure of the phyllosilicate mineral tuperssuatsiaite, including the positions of the hydrogen atoms in its unit cell, is determined for the first time by using first-principles solid-state methods. From the optimized structure, its infrared spectrum and elastic properties are determined. The computed infrared spectrum is in excellent agreement with the experimental spectrum recorded from a natural sample from Ilímaussaq alkaline complex (Greenland, Denmark). The elastic behavior of tuperssuatsiaite is found to be extremely anomalous and significant negative compressibilities are found. Tuperssuatsiaite exhibits the important negative linear compressibility phenomenon under small anisotropic pressures applied in a wide range of orientations of the applied strain and the very infrequent negative area compressibility phenomenon under external isotropic pressures in the range from 1.9 to 2.4 GPa. The anisotropic negative linear compressibility effect in tuperssuatsiaite is related to the increase of the unit cell along the direction perpendicular to the layers charactering its crystal structure. The isotropic negative area compressibility effect, however, is related to the increase of the unit cell dimensions along the directions parallel to the layers.

## Introduction

The compressibility is a fundamental material property measuring the change of the dimensions of a given material with respect to pressure. The isotropic negative linear compressibility (INLC)^1–14^ phenomenon is an important elastic anomaly found in solid materials in which one dimension increase upon hydrostatic compression. The closely related anisotropic linear compressibility phenomenon (ANLC)^9,10,12–14^ involves the increase of the dimensions of a material under the effect of a compressive pressure applied along a certain direction. The INLC phenomenon is measured in terms of the compressibility associated to certain dimension $\ell$, ${k}_{\ell }=1/\ell \cdot {(\partial \ell /\partial P)}_{P}$, where P is the external pressure. Conversely, the ANLC phenomenon is quantified by means of the volumetric compressibility along a certain direction, i.e., the directional derivative of the volume with respect to the pressure exerted in that direction, ${k}_{V}^{m}=1/V\cdot {(\partial V/\partial {P}_{m})}_{P}$. For space unconstrained solid materials which are thermodynamically and mechanically stable^5,15,16^ the total volume cannot increase under isotropic pressure and, therefore, the isotropic volumetric compressibility, ${k}_{V}=1/V\cdot {(\partial V/\partial P)}_{P}$, must be strictly positive. Counterexamples to the general statement of positivity of the isotropic volumetric compressibility have only been found for constrained systems^16^ and in the vicinity of material instabilities as phase transitions.^15^ However, in the case of anisotropic pressures there is not such limitation and the anisotropic volumetric compressibility ${K}_{V}^{m}$ can be negative.

Several possible different mechanisms explaining the onset of the negative linear compressibility (NLC) phenomenon have been found. These mechanisms were reviewed by Cairns and Goodwin.^8^ Some of the most important NLC mechanisms are the presence of ferroelastic instabilities, phonon instabilities, ferroelastic phase transitions and the anomalous mechanical behavior found in some materials displaying correlated polyhedral tilts and helical and wine-rack structural motifs. Recently, several distinct mechanisms have been encountered.^9–14^ For materials composed of structural elements as chains or sheets held together by means of van der Waals interactions,^9,10^ the application of pressure leads frequently to an anomalous increase of the interchain or intersheet distances leading to NLC effects. The anomalous mechanical behavior of chain structures has also been found in some materials with covalent-bonded chains.^14^ For uranyl squarate monohydrate,^12^ the NLC phenomenon appears to be a related with the peculiar behavior under pressure of the internal geometry of the uranium coordination polyhedra. In the case of the tridimensional structure of silver oxalate, the NLC effect is only due to the variation of the relative distances between the silver coordination polyhedra under pressure.^13^ The NLC anomaly in silver oxalate is the most extreme found up to date, not only because the magnitude of the negative compressibility is the largest found so far, but also because the range of external pressures in which the compressibility is negative is also the largest one. Finding the precise NLC mechanisms for a wide range of crystal structures is a very important matter because NLC is very frequently associated to specific structural motifs. Hence, structure-function relationships can be discovered allowing to anticipate the presence of interesting effects for specific structure types.^1,8,14^ An extremely infrequent particular case of the NLC phenomenon is the negative area compressibility (NAC) phenomenon.^11,17–21^ NAC is found for materials which expand along two directions under isotropic compression. Since the total volume should not increase, the dimension along the axis perpendicular to the plane formed by the two growing directions must decrease significantly to compensate the rise of the perpendicular area. Usually, the materials showing the NAC phenomenon, also exhibit negative thermal expansion.^17–19^ The negative compressibility phenomena can be found also for negative external pressures.^11,13,14^ In this case the material contracts under the effect of expansive pressures.

The NLC phenomena has an enormous number of important potential applications, such as the development of ultrasensitive pressure detectors, robust shock-absorbing composites, pressure-driven actuators, optical telecommunication cables, artificial muscles, next generation body armor and devices for biomedical applications.^2,3,9,22–24^ Synthetic and design approaches have been developed to obtain materials with improved mechanical performance.^4,9,25–27^ However, the number of natural and man-made NLC materials known so far is limited and, for these materials, the magnitude of the negative compressibility and the range of external pressures for which these phenomena are displayed are too small to be widely exploitable in practice.

Although, systematic studies of the mechanical properties of solid materials have been carried out,^1,4,28–30^ the extent of these studies is limited and many interesting materials have been left out. The case of organic materials is a clear example. The study of the negative mechanical phenomena in this kind of materials has been very scarce and recent studies^9–11,13,14^ point out that this omission constitutes a significant gap in the research of elastic anomalies. One of the main purposes of this study is to study the negative compressibility phenomena and the corresponding NLC mechanisms for an interesting inorganic natural material. This material is a natural iron and manganese-rich phyllosilicate mineral called tuperssuatsiaite,^31^ with composition ${\text{Na}}_{2}{\text{Fe}}_{2}\text{Mn}[{\text{Si}}_{8}{\text{O}}_{20}]{(\text{OH})}_{2}\cdot 6{\text{H}}_{2}\text{O}.$ In the macroscopic world, the elasticity of materials with empty spaces or air chambers are very interesting due to their elastic response and their applications for shock and acoustic attenuation. Intuition suggests that the elastic properties of highly porous crystalline materials should be investigated in detail, even although the response of materials at the nanometer scale resulting from the interaction between the atoms in their unit cells may differ from the expectations due to the quantum mechanical origin of these interactions. The elastic behavior of zeolites,^32–40^ carbon nanotubes,^3,41–46^ carbon honeycombs,^47^ elastomers^48^ and other porous materials^49–55^ is remarkable and has been studied at length due to its potential applicability. In this work, the elastic properties of tuperssuatsiaite mineral were studied because it possesses a porous crystal structure with empty cylindrical channels parallel to [001] crystallographic direction (or partially filled with water).

The crystal structure of tuperssuatsiaite is incompletely known^31^ because the determination of the positions of the hydrogen atoms in the corresponding unit cell was not possible from X-ray diffraction data. Hence, it was determined here using the first principles solid-state methodology. The methods employed in this paper have been successful in determining the full crystal structures of solid materials.^12,56,57^ The infrared spectrum of this mineral was then determined from the computed crystal structure and recorded from a natural mineral sample from Ilímaussaq alkaline complex, Greenland, Denmark. Since both spectra were in excellent agreement, a normal coordinate analysis of the theoretical vibrational data was used in order to assign all the bands of the experimental spectrum. Finally, the elastic properties of tuperssuatsiaite were also determined by means of large-scale first principles calculations. A large elastic response may be expected intuitively as a result of the compression in this kind of structure.

This paper is organized as follows. The methods employed in this work are described in the next Section. The optimized crystal structure, the experimental and theoretical infrared spectra and the computed elastic properties of tuperssuatsiaite are reported in the Results Section. These results are discussed in the fourth Section. The conclusions of this work are presented in the last section.

## Methods

### Experimental

**Figure 1:**
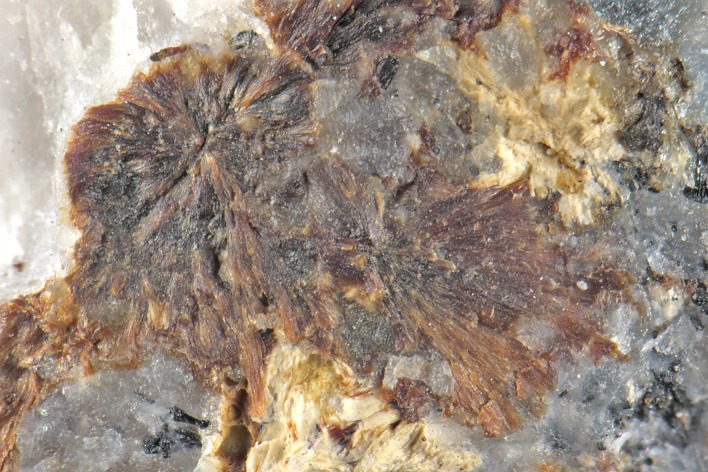
Tuperssuatsiaite brown radial aggregates from Ilímaussaq alkaline complex, Greenland, Denmark. The horizontal field-of-view is 4 mm.

The powder X-ray diffraction (PXRD) pattern of a natural mineral sample of tuperssuatsiaite from Ilímaussaq alkaline complex, Greenland (Denmark) was collected at room temperature on a Bruker D8 Advance diffractometer (National Museum, Prague) with a solid-state 1D LynxEye detector using ${\text{CuK}}_{\alpha }$ radiation and operating at 40 kV and 40 mA. The sample is shown in Fig. 1. In order to minimize the background signal, the powder samples were placed on the surface of a flat silicon wafer in acetone suspension. The powder pattern was collected using Bragg–Brentano geometry in the range of 2θ from 3 to 70°, in steps of 0.01° with a counting time of 20 seconds per step.

The Fourier-transform infrared (FTIR) spectrum of tuperssuatsiaite was recorded by the attenuated total reflection (ATR) method with a diamond cell on a Nicolet iS5 spectrometer. The spectrum was collected over the wavenumber range from 4000 to 390 cm^-1^. The spectrum was obtained by the co-addition of 32 scans with a resolution 4 cm^-1^ and a mirror velocity of 0.4747 cm/s. The spectra were co-added to improve the signal-to-noise ratio. All the spectral manipulations were carried out using Omnic 9 software (Thermo Scientific).

### First-principles solid-state methods

The crystal structure, infrared spectrum and mechanical properties of tuperssuatsiaite were modeled employing the Cambridge Serial Total Energy Program (CASTEP),^58^ a component of the Materials Studio program suite.^59^ The theoretical solid-state treatment employed in this work is based on Periodic Density Functional Theory using plane wave basis sets and pseudopotential functions to describe the internal atomic electrons.^60^ The computations were carried out using the Perdew-Burke-Ernzerhof (PBE) energy-density functional^61^ complemented with Grimme’s empirical dispersion correction.^62^ The specific pseudopotentials utilized were standard norm-conserving pseudopotentials^63^ from CASTEP package. The unit-cell parameters of tuperssuatsiaite and the associated atomic positions were fully optimized by means of the Broyden–Fletcher–Goldfarb–Shanno (BFGS) technique.^64^ A plane wave kinetic energy cut-off parameter of ε = 900 eV and a $k$-mesh^65^ of 2 $\times$ 1 $\times$ 4 were employed. These calculation parameters were chosen to attain a well converged crystal structure and energy (see Tables S.1 and S.2 of the Supplementary Information). The powder X-ray diffraction pattern of tuperssuatsiaite was generated from the computed crystal structure employing the software REFLEX included in Materials Studio package.^59^

The theoretical computation of the infrared spectrum of tuperssuatsiaite was performed by means of density functional perturbation theory (DFPT).^66,67^ The harmonic approximation of the interatomic force field was used for the calculation of the infrared vibrational frequencies and the corresponding band intensities. No scaling procedures were applied. These scaling procedures are frequently used to improve the computed infrared frequencies by correcting them empirically for the anharmonicity and remaining approximations used.^68^ However, the unscaled infrared frequencies and computed intensities obtained using the DFPT method for tuperssuatsiaite provided a consistent description of its infrared spectrum.

The stiffness tensor^69^ elements required to calculate the mechanical properties of tuperssuatsiaite and to study the mechanical stability of its crystal structure were determined using the finite deformation method.^70^ The unit cell volumes in the neighborhood of the equilibrium structure were computed by optimizing the crystal structure under sixteen different external pressures with values in the range −1.0 to 9.0 GPa. The computed lattice volumes and associated pressures were then adjusted to a 4^th^ order Birch-Murnahan equation of state^71^ in order to extract the derivatives of the bulk modulus with respect to pressure. Angel’s EOSFIT 5.2 code^72,73^ was employed for fitting the pressure-volume data to the selected equation of state. The structural optimizations under pressure were also performed using the BFGS method. The computation of the elastic matrix elements and all crystal structure optimizations performed in this work were carried out with stringent convergence tolerances in the variation of the total energy, maximum atomic force, maximum atomic displacement and maximum stress of 0.5 × 10^−5^ eV/atom, 0.01 eV/Å, 0.5 × 10^−3^ Å and 0.02  GPa, respectively. These convergence parameters correspond to the standard “hyperfine” optimization in CASTEP.

The use of special crystal structure prediction algorithms was not required to determine the full crystal structure of tuperssuatsiaite. The input structure for the crystal structure determination was the experimental structure (lacking the positions of the hydrogen atoms) reported by Camara *et al*.^31^ supplemented by initial values for the hydrogen atom positions. These initial positions were proposed according to a simple procedure. For a given water or hydroxyl oxygen atom the nearest oxygen atoms in the crystal structure were located. Then, two hydrogen atoms were placed along the lines linking the water oxygen atoms and two near oxygen atoms with the condition that the geometry of a water molecule is approximately attained. For the hydroxyl oxygen atoms only one hydrogen atom has to be introduced. The hydrogen atoms were put at a distance from the water or hydroxyl oxygen atoms of about 1.1 **Å**. All the possible structures resulting from the different initial positions of the hydrogen atoms were then fully optimized using the BFGS method. The optimization of these structures yielded several final structures but all of them except one were discarded using energy criteria. The positiveness of the energy hessian matrix for this structure was verified.

## Results

### Crystal structure

**Figure 2:**
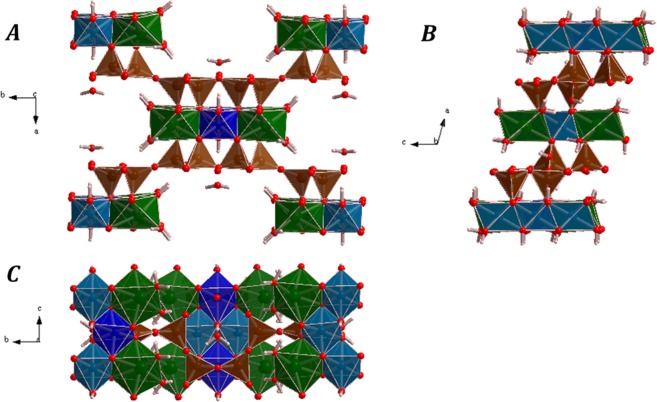
Computed crystal structure of tuperssuatsiaite: Views of the unit cell from (**A**) [001]; (**B**) [010]; (**C**) [100]. Color code: Si - Brown, Na - green, Fe- clear blue, Mn - dark blue, O - red, H - white.

The computed crystal structure of tuperssuatsiaite is shown in Fig. 2. The silicon atoms in the crystal structure of tuperssuatsiaite display, as usual, tetrahedral coordination (${\text{SiO}}_{4}^{4-}$). There are two symmetrically independent silicon atoms, Si1 and Si2, in its unit cell. The two independent silicon atoms occupy tetrahedral sites denoted as T1 and T2.^31^ The sodium, iron and manganese atoms possess octahedral coordination. There are one, two and two symmetrically independent sodium (Na), iron (Fe1 and Fe2) and manganese (Mn1 and Mn2) atoms, respectively. The octahedral sites occupied by the sodium atom are referred to as M3 and the iron and manganese atoms occupy partially the so-called M1 and M2 octahedral sites.^31^ The coordination sphere of sodium atom is composed of four oxygen ions and two water molecules, ${\text{NaO}}_{4}{({\text{H}}_{2}\text{O})}_{2}$ and the coordination structure of iron and manganese atoms is similar, their ligands being four oxygen and two hydroxyl anions (${\text{MO}}_{4}{(\text{OH})}_{2}$, where M = Fe, Mn).

**Figure 3:**
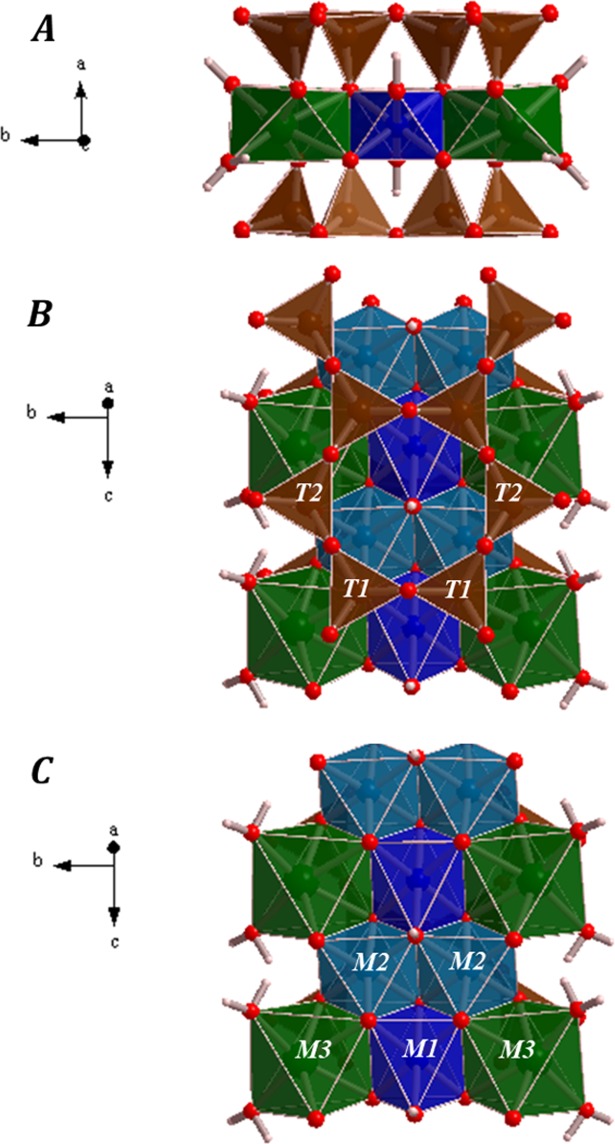
Images of an isolated octahedral band expanding along c direction in the crystal structure of tuperssuatsiaite mineral. The Subfigure (**A**) provides a view from [001]. In the subfigures (**B**) and (**C**), providing views from [100], the T1 and T2 tetrahedral sites and the M1, M2 and M3 octahedral sites are clearly distinguished, respectively. Color code: Si - Brown, Na - green, Fe - clear blue, Mn - dark blue, O - red, H - white.

The crystal structure of tuperssuatsiaite is composed of ribbons of silicate tetrahedra linked by bands of octahedra expanding in $c$ direction. The silicate ribbons and octahedral bands are displayed in detail in Fig. 3. The silicate ribbons are aligned parallel to the $c$ crystal axis and they are linked to form layers parallel to the (011) crystallographic plane. The octahedral bands are three octahedra wide and consists of alternating M3-M1-M3 and M2-M2 edge-sharing octahedra. In the unit cell of tuperssuatsiaite, there is one band structural unit at each vertex and one in the center. By repeating the unit cell of tuperssuatsiaite along $a$ or $b$ directions (see Fig. S1 of the Supplementary Information), the presence of a series of channels directed along $c$ direction is easily perceived. These channels, partially occupied by water molecules, are similar to those present in the crystal structure of the closely related mineral palygorskite and are important absorption sites.^74^ The M1 site, nearly fully occupied in tuperssuatsiaite, is equivalent to an octahedral site which is described as empty in palygorskite,^75,76^ although it is also reported to be partially occupied and forming a series between dioctahedral and trioctahedral palygorskite varieties.^74^ The computed unit cell parameters of tuperssuatsiaite are reported in Table S3 of the Supplementary Information. The computed crystal structure of tuperssuatsiaite is also given as a Supplementary Information in a file of CIF (Crystallographic Information File) format.

**Figure 4:**
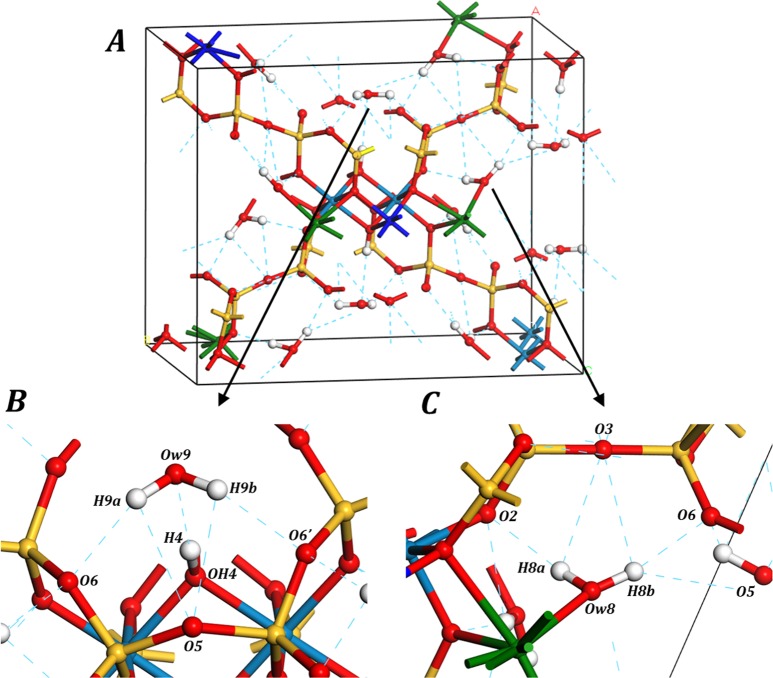
Hydrogen bond structure in tuperssuatsiaite mineral; (**A**) Image of the hydrogen bond network in the full unit cell; (**B**) Detailed image of the hydrogen bond structure surrounding a free water molecule; (**C**) Detailed image of the hydrogen bond structure surrounding a structural water molecule.

The hydrogen bonding structure in tuperssuatsiaite is shown in Fig. 4. There are twelve water molecules in the unit cell of tuperssuatsiaite (six per formula unit, ${\text{Na}}_{2}{\text{Fe}}_{2}\text{Mn}[{\text{Si}}_{8}{\text{O}}_{20}]{(\text{OH})}_{2}\cdot 6{\text{H}}_{2}\text{O}$) and only two of them are symmetry independent. Six of them may be described as “free water” because they are held to the structure by hydrogen bonding only. The remaining ones are described as “structural water” because they belong to the coordination structure of sodium atom.

There are 10 different hydrogen bond types in the crystal structure of tuperssuatsiaite. Five of these hydrogen bonds are associated to the free water molecules and the other five to the structural water molecules. These hydrogen bonds are illustrated in detail in Fig. 4(B) and Fig. 4(C), respectively. The computed hydrogen bond parameters are reported in Table S4. The hydrogen bonds associated to the free water molecules are Ow9-H9a···O6, Ow9-H9a···O5, Ow9-H9b···O5, Ow9-H9a···O6’ and OH4-H4···Ow9. The first and second hydrogen bonds are related by symmetry with the third and fourth ones. In the first two bonds, the donor oxygen atom is the free water oxygen atom and both bonds are mediated by the first water hydrogen atom, H9a. The third and fourth hydrogen bonds are identical to the first and second ones but are mediated by the second water hydrogen atom, H9b. The donor oxygen atom in the fifth hydrogen bond is the hydroxyl hydrogen atom OH4 and the acceptor oxygen atom is Ow9. The remaining five hydrogen bonds, associated to the structural water molecules are Ow8-H8a···O2, Ow8-H8a···O3, Ow8-H8b···O3, Ow8-H8b···O6 and Ow8-H8b···O5. The first two ones are mediated by H8a and the last three ones by H8b.

### Powder X-ray diffraction pattern

The Powder X-ray diffraction pattern of tuperssuatsiaite was measured experimentally and determined from the computed crystal structure. The experimental and theoretical PXRD patterns are compared in Fig. S2 of the Supplementary Information and as it can be seen the agreement is very satisfactory.

### Infrared spectrum

**Figure 5:**
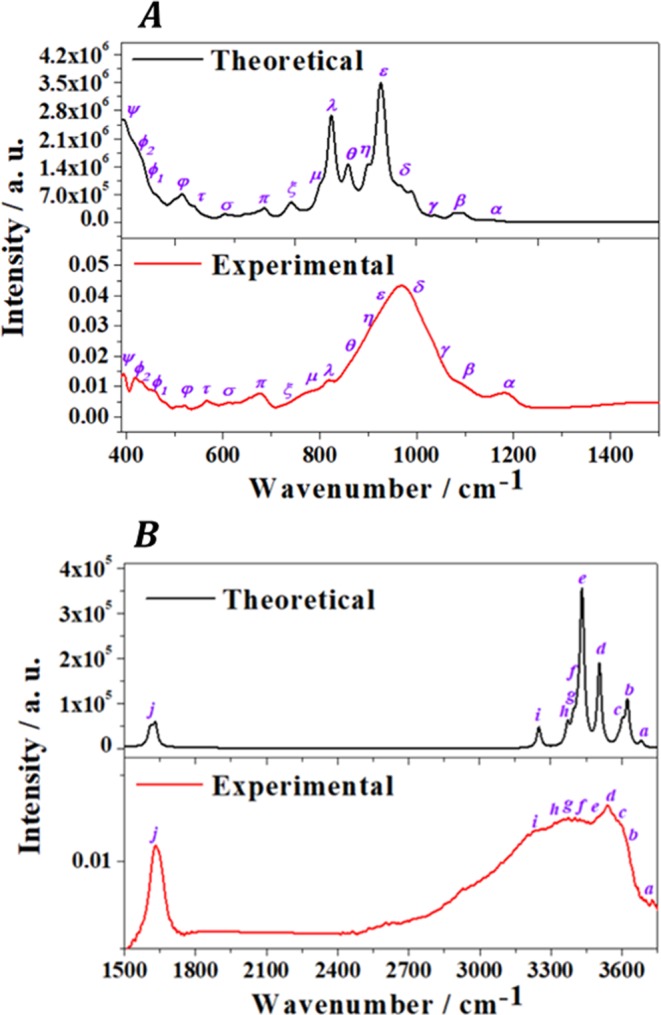
Experimental and theoretical infrared spectra of tuperssuatsiaite. (**A**) Region: 390–1500 ${\text{cm}}^{-1}$; (**B**) Region: 1500–3750 ${\text{cm}}^{-1}$.

The infrared spectrum of tuperssuatsiaite was recorded from a natural mineral sample from Ilímaussaq alkaline complex, Greenland (Denmark) and determined theoretically by means of density functional perturbation theory. As may be seen in Fig. 5, the experimental and theoretical spectra are in very good agreement. The bands in the experimental infrared spectrum are very broad and were resolved into components using the method described in a previous paper.^77^ The resolution of some selected experimental bands into single components is shown in Fig. S3. The band wavenumbers of both spectra along with the corresponding calculated intensities and assignments are given in Table S5 of the Supplementary Information. Images of the atomic motions in some infrared active vibrational modes of tuperssuatsiaite are provided in Fig. S4. The experimental infrared spectrum was analyzed in three different wavenumber regions: ($i$) $\text{OH}$ bond stretching vibration region from 3000 to 3750 cm^-1^ (Fig. 5(B)); ($ii$) The $\text{HOH}$ bending region from 1500 to 1700 cm^-1^ (see also Fig. 5(B)); and ($iii$) The low wavenumber region from 390 to 1500 cm^-1^ (Fig. 5(A)). The information of the specific infrared bands belonging to each spectral zone is given in different sections of Table S5.

### Elastic properties

The elasticity tensor of tuperssuatsiaite was computed using the finite deformation method. The matrix representation of the elastic tensor is given in Table S6 of the Supplementary Information. Since the crystal structure was determined using P1 triclinic symmetry, all the elastic constants of tuperssuatsiaite were non-vanishing.^69,78^ The mechanical stability of the crystal structure of tuperssuatsiaite was studied from the computed elasticity matrix. The mechanical stability of a given crystal structure requires that its elasticity matrix is positive definite, i.e., that all its eigenvalues be positive.^79,80^ The elasticity matrix was diagonalized numerically and a single eigenvalue was found to be negative. This means that tuperssuatsiaite is mechanically unstable. The instability of a crystal structure anticipates the presence of large structure deformations under the application of small pressures which, in extreme cases, lead to pressure induced phase transitions. In the present case, as shown below, the application of pressure leads to noticeable elastic anomalies.

The mechanical properties of polycrystalline aggregates of tuperssuatsiaite were computed from the calculated elastic constants using the schemes of Voigt,^81^ Reuss^82^ and Hill.^83^ The Voigt approach gave the best agreement between the computed bulk modulus with the single crystal bulk modulus determined from the equation of state (EOS) of tuperssuatsiaite. The computed mechanical properties of tuperssuatsiaite in the Voigt approximation are given in Table S7. The computed bulk modulus is 76.8 ± 4.1 GPa, which compares very well with the value derived from the EOS, 76.7 ± 4.3 GPa. The computed ductility index is $D$ = 1.95 and, therefore, tuperssuatsiaite is ductile because $D$ > 1.75^84,85^. Tuperssuatsiaite is a hard mineral since the calculated Vickers hardness is $H$ = 4.79^86^. As can be seen in Table S7, the universal anisotropy index^87^ of tuperssuatsiaite is ${A}^{U}$ = 3.16 which is quite large.

**Figure 6:**
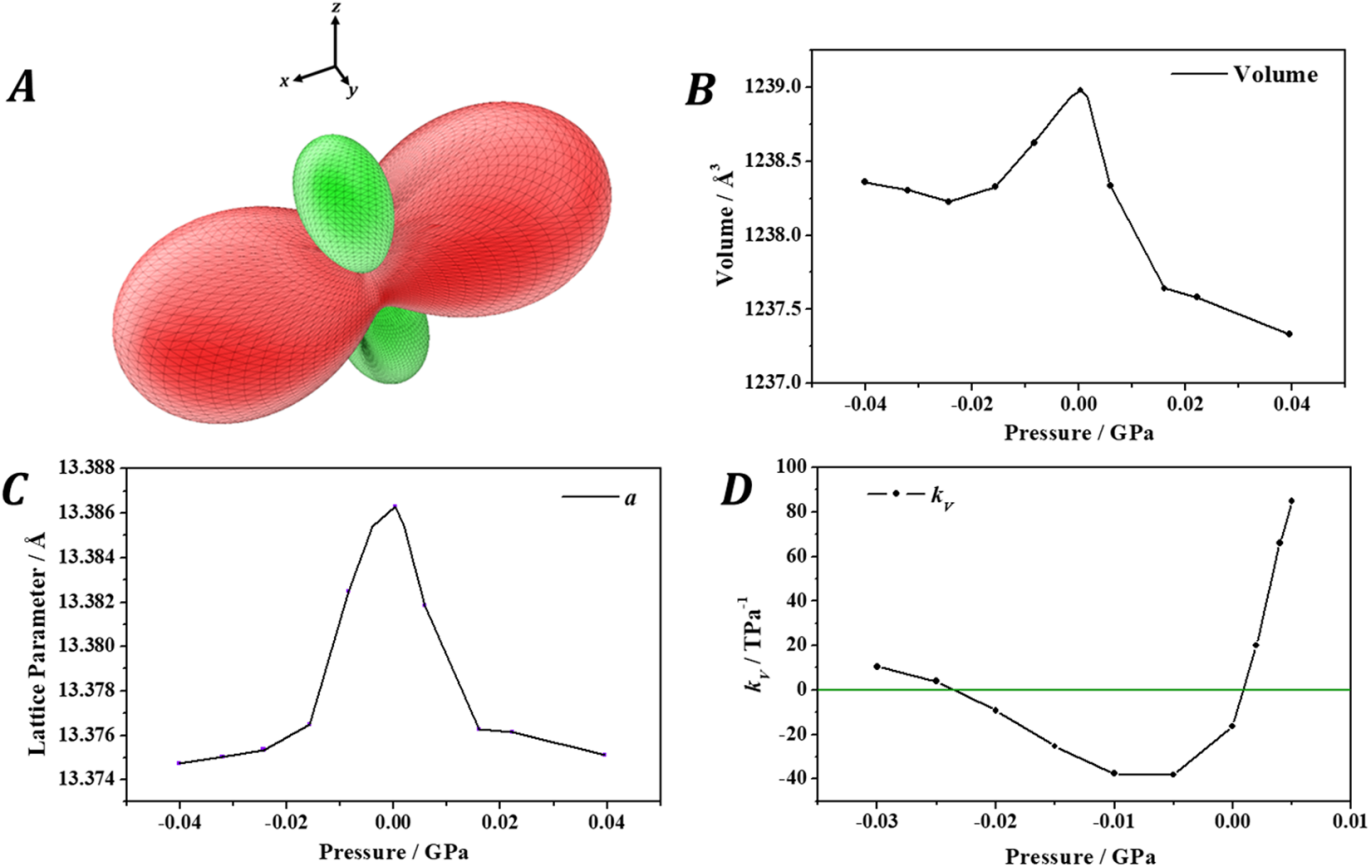
Tuperssuatsiaite under anisotropic pressure: (**A**) Computed compressibility of tuperssuatsiaite as a function of the orientation of the applied strain. The positive and negative values are displayed in green and red, respectively; (**B**) Calculated unit-cell volume; (**C**) $a$ lattice parameter; and (**D**) volumetric compressibility as a function of the external pressure applied in the direction of minimum Poisson’s ratio.

Since large elastic anisotropies are strongly correlated with large differences between the values of the maximum and minimum Poisson’s ratios when all possible directions of the applied strain are considered^88,89^, tuperssuatsiaite was a good candidate to present negative values of the Poisson’s ratio. Besides, since negative values of the Poisson’s ratio are frequently accompanied with negative values of the compressibility^9–14^, the dependence of the Poisson’s ratio and compressibility of tuperssuatsiaite on the orientation of the applied strain was investigated. Small negative values of the Poisson’s ratio were indeed found, the minimum value being ${\nu }_{min}$ = −0.22 for an strain applied along the direction ${U}_{\nu }^{min}$ = (0.17, 0.81, 0.33). More importantly, an extremely anomalous behavior of the compressibility was found. As can be observed in Fig. 6(A), tuperssuatsiaite exhibits large negative values of the compressibility for a wide range of orientations of the applied strain. In order to study the anisotropic NLC effect in tuperssuatsiaite in detail, its crystal structure was optimized under the effect of different values of the external pressure applied along the direction of minimum Poisson’s ratio using the same methods and convergence parameters as those used to determine the equilibrium crystal structure. The computed unit cell volumes and compressibilities under the effect of these pressures are shown in Fig. 6(B,D). The computed values of the compressibilities are also given in Table S8.

**Figure 7:**
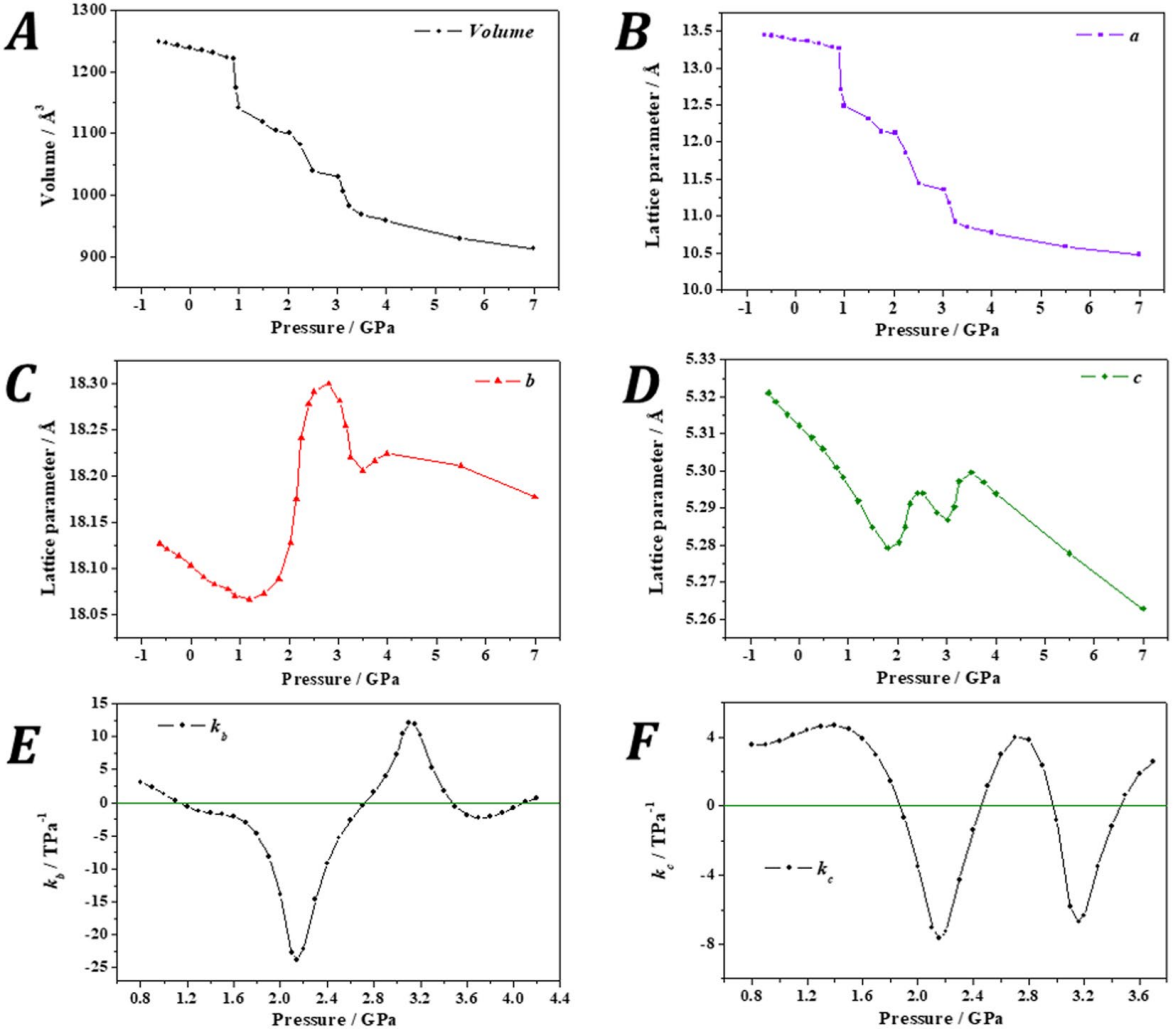
Calculated unit cell volume (**A**), lattice parameters $a$ (**B**), $b$ (**C**) and $c$ (**D**), and compressibilities along $b$ (**E**) and $c$ (**F**) directions of tuperssuatsiaite as a function of the applied external isotropic pressure.

The mechanical behavior of tuperssuatsiaite under the effect of isotropic pressure was also investigated. The computed unit cell volume and lattice parameters under the effect of different isotropic pressures are shown in Fig. 7. The computed compressibilities along *b* and *c* directions are displayed in Fig. 7(E,F), respectively, and given in Table S9.

## Discussion

### Crystal structure

As can be seen in Table S3, the computed and experimental crystal structures are in good agreement. The difference between the calculated and experimental volume and density reported by Cámara *et al*.^31^ is only about 3.3%. The main source for the difference between the computed and experimental crystal structures should be the different temperatures at which these crystal structures were determined. The computed structure corresponds to the temperature of 0 K and the experimental one to room temperature. These differences could be reduced to a large extent if experimental measurements of the crystal structure at low temperatures were performed.

The final structural calculations of Cámara *et al*.^31^ were performed with only one species (Fe) at the M1 and M2 sites and neglecting the partial occupancies of Fe and Mn in these sites. In the present work, the partial occupancy of these octahedral sites was taken into account in all the computational works. This feature led to the requirement of performing all the calculations with the space symmetry constraints relaxed (the monoclinic C2/m unit cell was relaxed to P1 symmetry) which caused that the calculations were very expensive. As a result, the computed geometries of the symmetry equivalent atoms in the original monoclinic structure were slightly different. The geometric data provided below correspond to the values averaged over all monoclinic equivalent sites. The two distinct Si atoms have experimental average Si−O distances of 1.615 and 1.610 Å, respectively.^31^ The computed average Si−O distances, 1.637 and 1.628 Å, agree very well with the experimental values, the differences being about 0.02 Å. The Na atoms have an average Na−O distance of 2.406 Å, which compares also well with the theoretical value of 2.347 Å. The iron and manganese atoms occupying partially the M1 and M2 sites have experimental average metal−oxygen distances of 2.064 and 2.038 Å,^31^ respectively. The computed values of these average distances for the M1 and M2 sites are 1.984 and 1.946 Å for the sites occupied by iron atoms and 1.991 and 1.981 Å for the sites occupied by the manganese atoms. Thus, the metal-oxygen distances are only slightly larger for the manganese atoms.

The oxygen atoms associated to the free and structural water molecules in the unit-cell of tuperssuatsiaite are denoted as Ow9 and Ow8, respectively, in the labelling convention used by Cámara *et al*.^31^. The computed optimized positions of the oxygen atoms in the free water molecules were found to be slightly different from that reported by Cámara *et al*.^31^. In fact, these authors noted the presence of a residual maximum in the difference-Fourier map located in the tuperssuatsiaite channels which was attributed either to the disordering of water molecules within the channels or to a partial occupancy of the water oxygen atom site. The position reported by Cámara *et al*.^31^ for Ow9 oxygen atom leads to water molecules within the channels which are not linked to the structure by hydrogen bonding (the distance of the water oxygen atom to the nearest oxygen atom within the structure is 3.5 Å). Therefore, it is possible that the experimental structure at room temperature should be complemented with additional water molecules occupying partially their positions and providing hydrogen bonding between the basic structure and the water molecules within the tuperssuatsiaite channels. The present calculations provided the minimum-energy positions of all the atoms in tuperssuatsiaite with six water molecules per formula unit at zero temperature without ambiguities.

### Powder X-ray diffraction pattern

Table S10 of the Supplementary Information provides a detailed comparison of the positions of the twenty-one most intense reflections [*hkl*] in the computed and experimental PXRD patterns of tuperssuatsiaite. The differences in these positions is quite small, the largest deviations being observed for the [4 0 0], [5 1 0] and [−7–5 1] reflections, that is, those having larger *h* indices. This reflects that the difference in the computed *a* unit-cell parameter (the length of the unit cell along the direction perpendicular to the layers) with respect to the corresponding experimental^31^ value is larger than in the other parameters (see Table S3). Since the computed crystal structure corresponds to zero temperature, the thermal expansion of the unit cell from zero to room temperature should increase the value of this lattice parameter providing a better agreement with the experimental PXRD patten. The thermal expansion of the interlayer space in phyllosilicates with empty or partially filled interlayer spaces is usually quite significant.

### Infrared spectrum

As can be noticed from Fig. 5 and Table S5, the infrared spectrum of tuperssuatsiaite was faithfully reproduced using the first principles methodology. This gives additional support to the computed crystal structure. Furthermore, since the bands in the experimental and theoretical spectra showed a very high degree of consistence, a normal coordinate analysis of the theoretical spectrum was carried out in order to assign all the bands in the observed spectrum to specific atomic vibrational motions (Table S5). All the bands in the spectral region from 3000 to 3750 cm^−1^ (denoted as bands *a* to *i*) are attributed to OH bond stretching vibrations. Likewise, in the region from 1500 to 1750 cm^−1^, the four sub-bands of band *j* (see Table S5 and Fig. S3(B)) are ascribed to water bending vibrations. The bands of the low wavenumber region from 390 to 1500 cm^−1^ (bands *α* to *ψ*) can be assigned to combinations of SiO, FeO and MnO bond stretching vibrations, FeOH, NaOH, SiOSi and FeOSi bending vibrations, OSiO wagging and SiOSi rocking vibrations, water librations and hydroxyl translations. The precise assignment for each band is given in Table S5 and some representative examples of the atomic vibrational motions in some infrared active vibrational normal modes of tuperssuatsiaite are displayed in Fig. S4.

### Elastic behavior

Figure 6(A) shows that tuperssuatsiaite has an extremely anomalous mechanical behavior under anisotropic pressure because it exhibits negative compressibilities for a wide range of orientations of the applied strain. Taking as an example of orientation the direction of minimum Poisson’s ratio (see Fig. 6(B,D)), the unit cell volume of tuperssuatsiaite increases under anisotropic compression from P = −0.023 to P = 0.002 GPa, the minimum compressibility being ${k}_{V}^{{\nu }_{min}}$ = −40.0 ${\text{TPa}}^{-1}$ at P = −0.007 GPa (Table S8). The deformation of the crystal structure of tuperssuatsiaite in this pressure region was analyzed and no significant changes in the interatomic distances and angles were found due to the small magnitude of the applied pressures. The most significant change found was the increase of the $a$ lattice parameter (Fig. 6(C)) caused by the increase of the vertical dimension of the channels of tuperssuatsiaite (Fig. S1).

Under isotropic pressure, the mechanical behavior is also anomalous. As shown in Fig. 7(A), the unit cell volume of tuperssuatsiaite decreases suddenly at P = 0.9 GPa. The unit volume decreases by nearly 48 Å^3^ from P = 0.89 to P = 0.93 GPa (4.1%). This reduction results almost completely from the drastic variation of the *a* lattice parameter (Fig. 7(B)), whose value changes from 13.27 to 12.70 Å (decreases by 4.2%). The *b* lattice parameter shows a large increase from P = 1.12 to P = 2.72 GPa and a smaller one from P = 3.48 to P = 4.09 GPa (Fig. 7(C)). Thus, in these two pressure ranges, the compressibility, *k*_*b*_ = −1/*b* (∂*b*/∂*P*)_*p*_, is negative (Fig. 7(E)). The minimum value of the compressibility along *b* direction is *k*_*b*_ = −23.92 TPa^−1^at P = 2.15 GPa (Table S9). Similarly, the *c* lattice parameter increases from 1.87 to 2.45 GPa and from 2.98 to 3.46 GPa (Fig. 7(D)), the minimum value of the compressibility, *k*_*c*_ = −1/*c* (∂*c*/∂*P*)_*p*_, being *k*_*c*_ = −7.70 TPa^−1^ at P = 2.16 GPa. Thus, tuperssuatsiaite exhibits the negative area compressibility phenomenon from 1.87 to 2.45 GPa because the compressibilities along *b* and *c* directions are simultaneously negative in this pressure range. The pressures at which the compressibilities along *b* and *c* directions are minimum are almost the same (P ∼ 2.15 GPa). The computed crystal structure at P = 2.509 GPa is given as a Supplementary Information in a file of CIF format.

**Figure 8:**
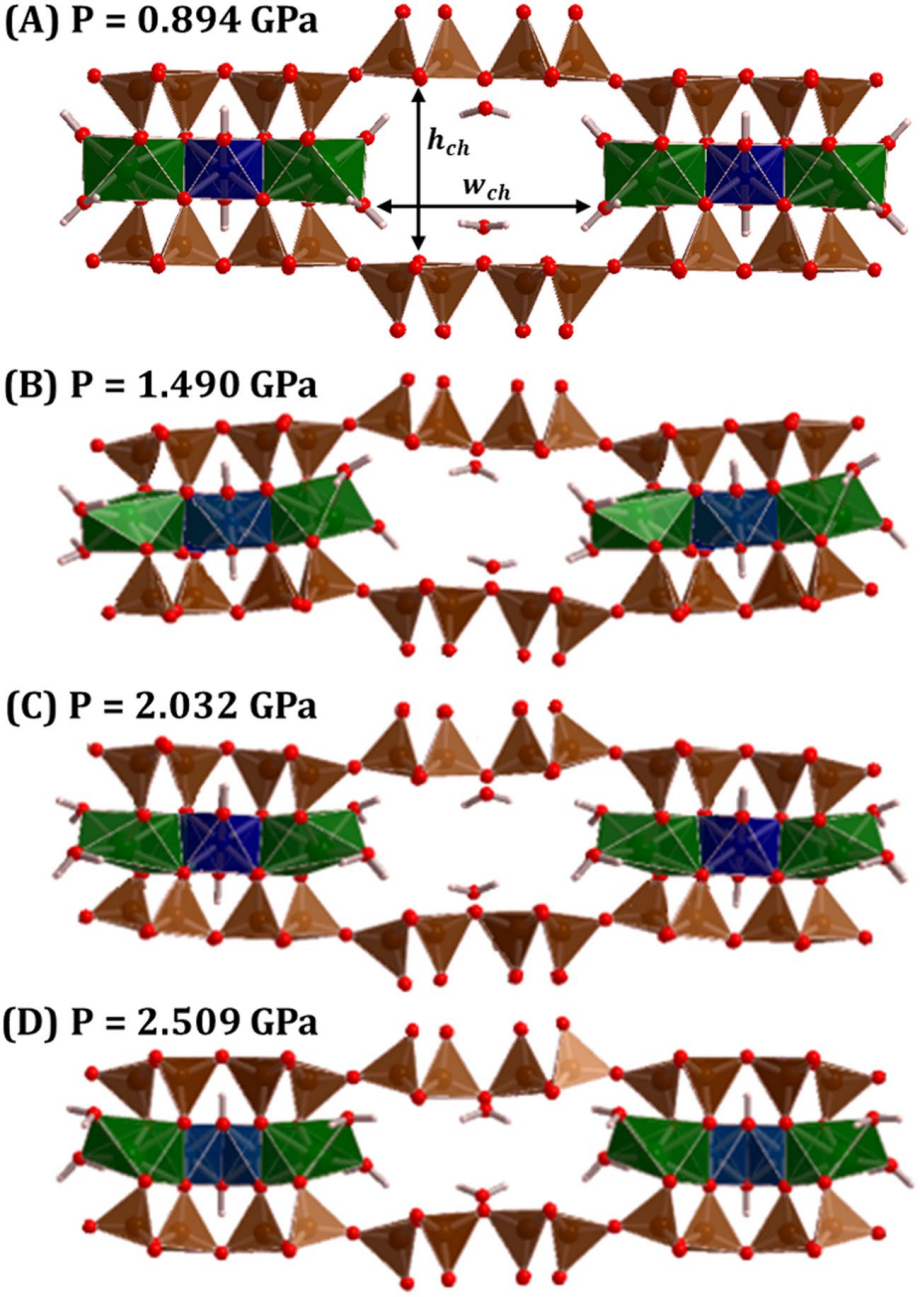
The structure of tuperssuatsiaite under isotropic pressure. View of two octahedral bands and an intermediate channel at different pressures: (**A**) P = 0.894 GPa; (**B**) P = 1.490 GPa; (**C**) P = 2.032 GPa; (**D**) P = 2.509 GPa. The meaning of the width and height (${w}_{ch}$ and ${h}_{ch}$) of a channel is illustrated in the subfigure (A). The values of ${w}_{ch}$ and ${h}_{ch}$ for each pressure are given in Table S11.

Figure 8 shows two octahedral bands and an intermediate channel in the crystal structure of tuperssuatsiaite for four different isotropic pressures (P = 0.894, 1.490, 2.032 and 2.509 GPa). The most important variations of the crystal structures associated to these pressures are given in Table S11. At P = 0.894 GPa (Fig. 8(A)) the structure is topologically identical to the structure at zero pressure (Fig. 2). However, as seen in the previous paragraph, a drastic reduction of *a* lattice parameter and unit-cell volume occurs at a pressure of about 0.90 GPa. As it may be observed in Fig. 8(B), the main structural changes originating this drastic reduction are the strong distortion of the sodium coordination polyhedra and the curving of the silicate layers. The O1−Na−O9 interatomic angle is reduced by nearly 20 degrees with respect to its value at P = 0.894 GPa (see Table S11) and, therefore, the height of sodium octahedra becomes strongly reduced. Besides, the width and the height of the channels (*w*_*ch*_ and *h*_*ch*_) decrease sharply. The crystal structure is topologically the same after this drastic structure change. However, as the pressure increases, while the height of the channels continues decreasing, their width increases extraordinarily leading to the increase of the *b* lattice parameter from 1.12 to 2.72 GPa. The *c* lattice parameter also increases from 1.87 to 2.45 GPa and, therefore, the channels become larger as the pressure increases.

## Conclusions

The determination of the positions of the hydrogen atoms in the crystal structure of tuperssuatsiaite was not possible from X-ray diffraction data and they were determined by using first-principles solid-state methods for the first time. The computed and experimental (without the positions of the hydrogen atoms) structural data were in good agreement. The infrared spectrum of tuperssuatsiaite was recorded from a natural sample from Ilímaussaq alkaline complex (Greenland, Denmark) and determined theoretically from the computed crystal structure. The good agreement between both spectra provided further support for the optimized crystal structure and allowed for the rigorous assignment of the bands in the infrared spectrum.

The elastic properties of tuperssuatsiaite were also determined from the energy optimized structure. It was found that this mineral displays significant elastic anomalies under the effect of external anisotropic and isotropic pressures. Tuperssuatsiaite exhibits the negative linear compressibility phenomenon under small anisotropic pressures applied in a wide range of orientations of the applied strain. The value of minimum compressibility for external pressures applied along the direction of minimum Poisson’s ratio is ${k}_{V}^{{\nu }_{min}}$ = −40.0 ${\text{TPa}}^{-1}$ at P = −0.007 GPa. Furthermore, tuperssuatsiaite exhibits the negative area compressibility phenomenon under external isotropic pressures in the range from 1.9 to 2.4 GPa. The compressibilities along $b$ and $c$ directions are minimum at almost the same external pressure, P ∼ 2.15 GPa. At this pressure, the compressibilities are *k*_*b*_ = −23.9 TPa^−1^ and *k*_*c*_ = −7.7 TPa^−1^. The use of accurate non-empirical quantum mechanical first principles methods allows not only for the computation of the values of the compressibilities but also a direct, rigorous and easy interpretation of the deformation of the crystal structures under pressure. While the anisotropic negative linear compressibility effect is related to the increase of the unit-cell along the direction perpendicular to the tuperssuatsiaite layers, the isotropic negative area compressibility results from the increase of the unit cell dimensions along the directions parallel to the layers. Thus, this mineral displays significant elastic anomalies for both anisotropic and isotropic pressures which result from completely different mechanisms. The elastic anomalies in tuperssuatsiaite are closely related to its porous crystal structure having empty or partially filled channels. Under isotropic pressures the silicate layers become curved towards the channels and, from 1.9 to 2.4 GPa, while the height of the channels decreases, their width and length increase substantially. While the mechanical properties of other important porous materials have been studied in detail,^32–55^ this study suggests that the mechanical properties of additional mineral species belonging to the group of phyllosilicate minerals with modulated layers^90^ or synthetic materials having microscopic structures similar to the structure of tuperssuatsiaite should be also investigated. The study of the variation of the mechanical properties of these materials as a function of the partial occupation of the channels with water could also be very interesting.

## Supplementary information

Supplementary Information.
