# Supplementary material for: Crystal Structure, Infrared Spectrum and Elastic Anomalies in Tuperssuatsiaite
Source: Sci Rep. 2020 May 5;10:7510. doi: 10.1038/s41598-020-64481-8 (PMC7200798; doi:10.1038/s41598-020-64481-8)
Supplement: Supplementary file 1 — Supplementary Information. [file 41598_2020_64481_MOESM1_ESM.pdf]

SUPPLEMENTARY INFORMATION

Crystal Structure, Infrared Spectrum and Elastic Anomalies in Tuperssuatsiaite

Francisco Colmenero,[1] Jiří Sejkora[2] and Jakub Plášil[3]*

[1]Instituto de Estructura de la Materia (IEM-CSIC), 28006 Madrid, Spain

[2]Mineralogicko-petrologické oddělení, Národní museum, 193 00 Praha 9, Czech Republic

[3]Institute of Physics ASCR, 182 21 Praha 8, Czech Republic

[*] Email: francisco.colmenero@iem.cfmac.csic.es

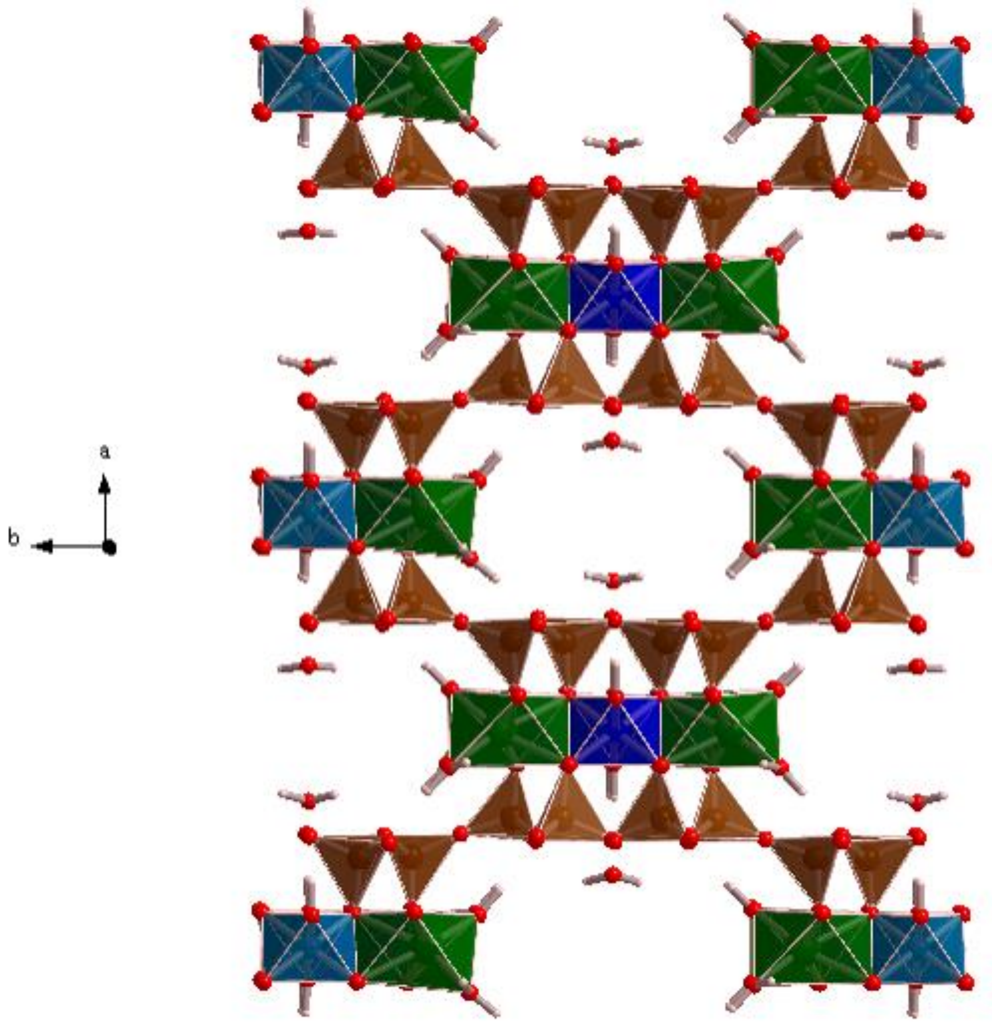

Fig. S1. Image of a 2 × 1 × 1 supercell of tuperssuatsiaite from [001] crystallographic direction.

Table S1. Total energies and unit-cell parameters of tuperssuatsiaite obtained using different values of the kinetic energy cutoff, ε , and a k-mesh of 2×1×4.

$\boldsymbol{\varepsilon}$(eV)	$\boldsymbol{E_{TOT}}$(eV)	$\boldsymbol{a}$(Å)	$\boldsymbol{b}$(Å)	$\boldsymbol{c}$(Å)	$\boldsymbol{\beta}$ (deg)	$\boldsymbol{Vol}$(Å^3)
800	-44782.2338	13.4043	18.1103	5.3144	105.6987	1241.9669
850	-44783.2028	13.3922	18.1022	5.3127	105.7322	1239.6847
900	-44783.5906	13.3863	18.1052	5.3113	105.7417	1238.9805
950	-44783.7214	13.3774	18.1013	5.3108	105.7598	1237.6587

Table S2. Total energies and unit-cell parameters of tuperssuatsiaite obtained using a kinetic energy cutoff of ε = 900 eV and different k-meshes.

$\boldsymbol{k}$-mesh ($\Delta\boldsymbol{k}$)	$\Delta\boldsymbol{k}$ (1/Å)	$\boldsymbol{E_{TOT}}$(eV)	$\boldsymbol{a}$(Å)	$\boldsymbol{b}$ (Å)	$\boldsymbol{c}$ (Å)	$\boldsymbol{\beta}$ (deg)	$\boldsymbol{Vol}$(Å^3)
1×1×2	0.08	-44783.693	13.3731	18.1038	5.3111	105.7680	1237.4528
1×1×3	0.06	-44783.582	13.3794	18.0988	5.3123	105.7279	1238.2356
2×1×4	0.05	-44783.591	13.3863	18.1052	5.3113	105.7417	1238.9805
2×1×5	0.04	-44783.585	13.3813	18.1025	5.3117	105.7284	1238.5041
2×2×5	0.035	-44783.288	13.3790	18.0754	5.3115	105.6095	1237.0956

Table S3. Computed unit-cell parameters, volume and density of mineral tuperssuatsiaite. The computed and experimental data correspond to zero and room temperature, respectively.

Parameter	a (Å)	b (Å)	c (Å)	α (deg)	β (deg)	γ (deg)	Vol. (Å^3)	ρ (g/cm^3)
**DFT**	13.3863	18.1052	5.3113	90.0	105.7417	90.0	1238.9805	2.411
**Exp. [1]**	14.034	17.841	5.265	90.0	103.67	90.0	1280.9166	2.332

Table S4. Computed hydrogen bond (O1-H···O2) parameters in tuperssuatsiaite (distances are given in Å and angles in degrees)

Hydrogen bond		d(O1···O2)	d(H···O2)	α(O1−H···O2)
**Water molecule**	**O1−H···O2**			
**H9a−Ow9−H9b**	Ow9−H9a···O6	2.88	2.13	133
	Ow9−H9a···O5	2.95	2.69	100
	Ow9−H9b···O5	2.95	2.69	100
	Ow9−H9b···O6’	2.88	2.13	133
	Ow9−H9b···O5	2.95	2.69	100
**H8a−Ow9−H8b**	Ow8−H8a···O2	2.85	1.95	152
	Ow8−H8a···O3	3.07	2.65	106
	Ow8−H8b···O3	3.07	2.70	103
	Ow8−H8b···O6	2.95	1.99	168
	Ow8−H8b···O5	3.28	2.65	123

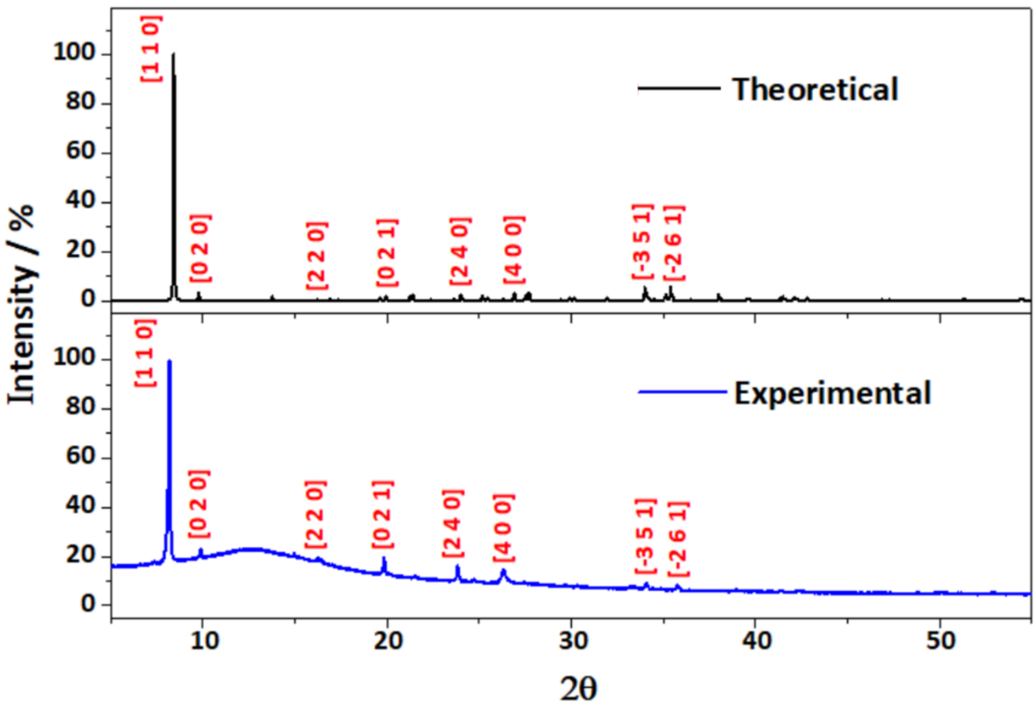

Fig. S2. Experimental and computed powder X-ray diffraction patterns of tuperssuatsiaite with CuK_α radiation (λ = 1.540598 Å).

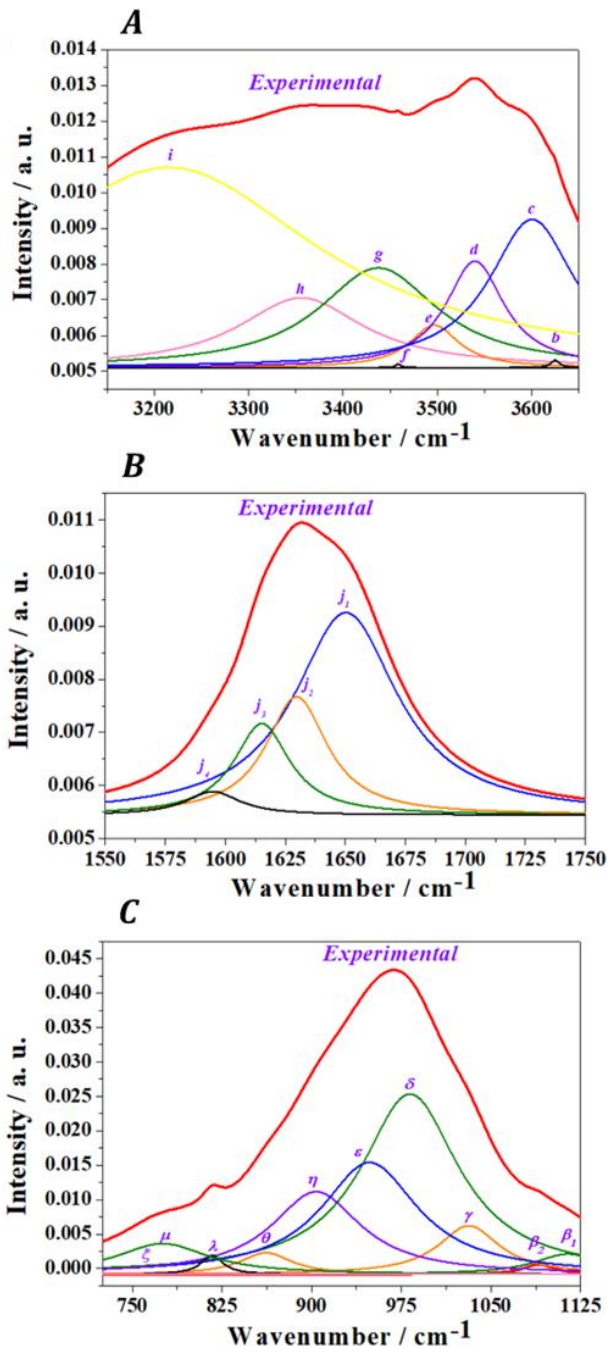

Fig. S3. Resolution of the composite bands in the experimental infrared spectrum of tuperssuatsiaite into single band contributions (A) Region: 3150-3650 cm^{-1}; (B) Region: 1550-1750 cm^{-1}; (C) Region: 725-1125 cm^{-1}.

Fig. S4. The atomic motions associated to some infrared active vibrational normal modes of tuperssuatsiaite. Color code: Si - Brown, Na – pink, Fe- clear blue, Mn - dark blue, O - red, H - white.

- Mode $\nu = 3506\ cm^{-1}$ – $\nu(OH)$ – OH bond stretching.

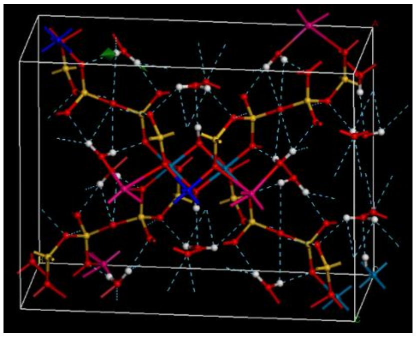

- Mode $\nu = 3431\ cm^{-1}$ – $\nu(OH)$ – OH bond stretching.

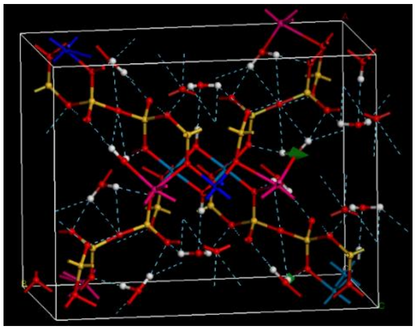

- Mode $\nu = 1632\ cm^{-1}$ – $\delta(HOH)$ – HOH bending.

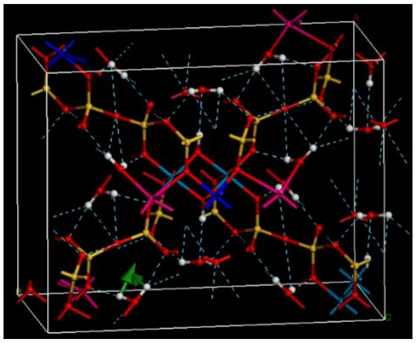

- Mode $\nu = 1153\ cm^{-1}$ – $\nu(SiO)$ – SiO bond stretching.

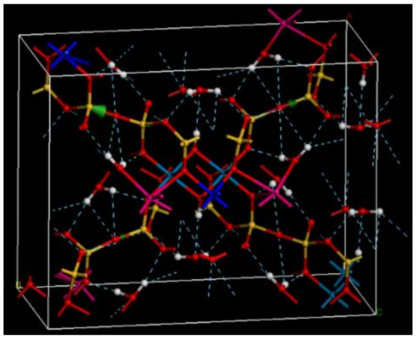

- Mode $\nu = 1105\ cm^{-1}$ – $\nu(SiO) + \delta(FeOH)$ – SiO bond stretching and FeOH bending.

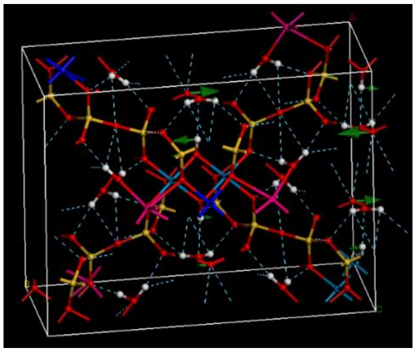

- Mode $\nu = 1039\ cm^{-1}$ – $\nu(SiO)$ – SiO bond stretching.

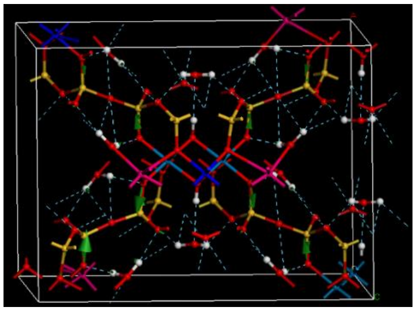

- Mode $\nu = 992\ cm^{-1}$ – $\delta(FeOH)$ – FeOH bending.

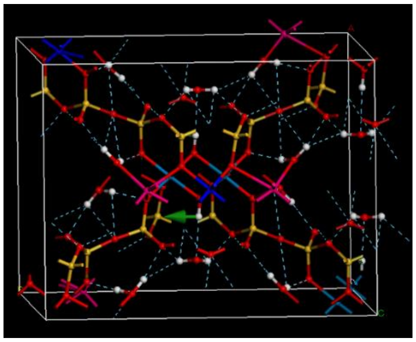

- Mode $\nu = 968\ cm^{-1}$ – $\nu(SiO) + \delta(FeOH)$ – SiO bond stretching and FeOH bending.

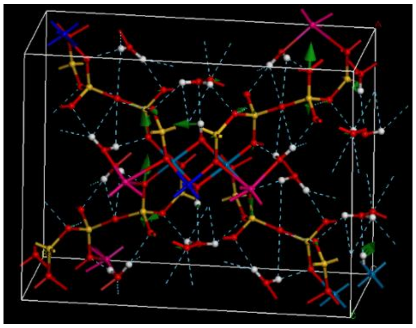

- Mode $\nu = 925\ cm^{-1}$ – $\delta(FeOH)$ – FeOH bending.

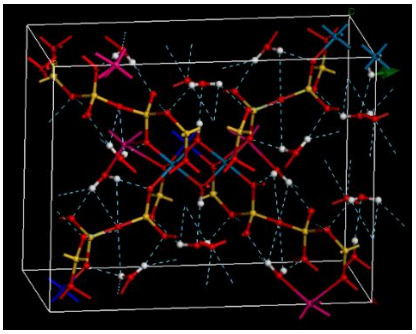

- Mode $\nu = 824\ cm^{-1}$ – $\nu(SiO) + \delta(FeOH)$ – SiO bond stretching and FeOH bending.

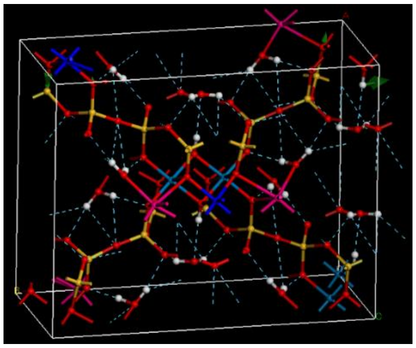

- Mode $\nu = 803\ cm^{-1}$ – $\nu(SiO) + \delta(FeOH) + l(H_2O)$ – SiO bond stretching, FeOH bending and water librations.

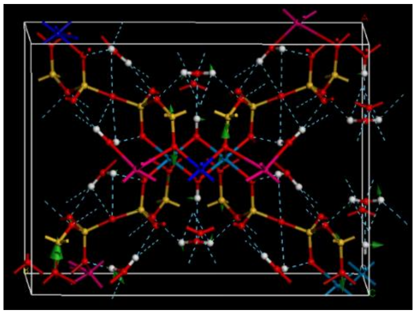

- Mode $\nu = 799\ cm^{-1}$ – $\nu(SiO) + l(H_2O)$ – SiO bond stretching and water librations.

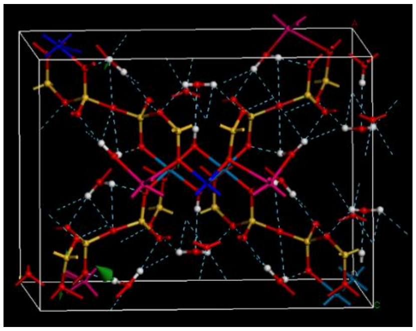

- Mode $\nu = 742\ \mathrm{cm}^{-1}$ – $l(H_2O)$ – Water librations.

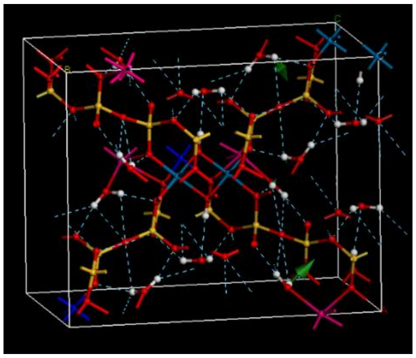

- Mode $\nu = 688\ \mathrm{cm}^{-1}$ – $\nu(\mathrm{SiO}) + \rho(\mathrm{SiOSi}) + \delta(\mathrm{FeOH}) + l(H_2O)$ – SiO bond stretching, SiOSi rocking, FeOH bending and water librations.

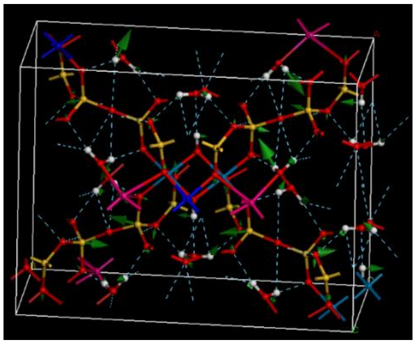

- Mode $\nu = 645\ \mathrm{cm}^{-1}$ – $\nu(\mathrm{SiO}) + \omega(\mathrm{OSiO}) + l(H_2O)$ – SiO bond stretching, OSiO wagging and water librations.

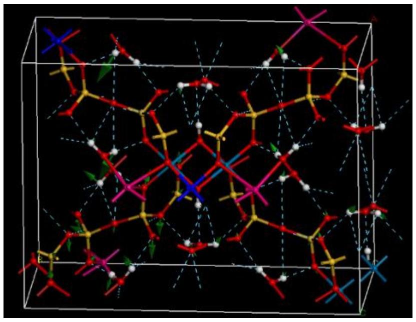

- Mode $\nu = 603\ \mathrm{cm}^{-1}$ – $\nu(\mathrm{SiO}) + \omega(\mathrm{OSiO}) + \rho(\mathrm{SiOSi}) + \delta(\mathrm{FeOH}) + l(H_2O)$ – SiO bond stretching, OSiO wagging, SiOSi rocking, FeOH bending and water librations.

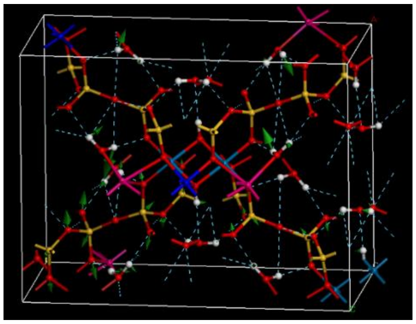

- Mode $\nu = 515\ \text{cm}^{-1}$ – $\delta(\text{FeOSi}) + \delta(\text{SiOSi}) + l(\text{H}_2\text{O})$ – FeOSi and SiOSi bending and water librations.

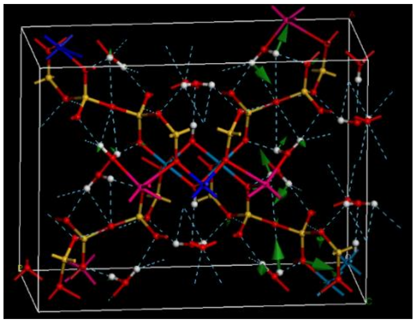

- Mode $\nu = 437\ \text{cm}^{-1}$ – $\nu(\text{FeO}) + \delta(\text{NaOH}) + \text{T}(\text{OH}) + l(\text{H}_2\text{O})$ – FeO bond stretching, NaOH bending, hydroxyl translation and water librations.

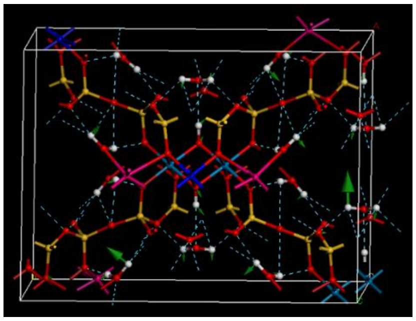

- Mode $\nu = 408\ \text{cm}^{-1}$ – $\delta(\text{NaOH}) + l(\text{H}_2\text{O})$ – NaOH bending water librations.

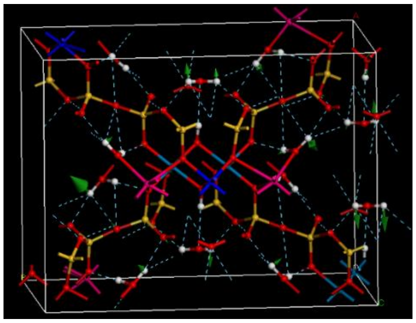

- Mode $\nu = 398\ \text{cm}^{-1}$ – $\nu(\text{MnO}) + \delta(\text{FeOH}) + \text{T}(\text{OH}) + l(\text{H}_2\text{O})$ – MnO bond stretching, FeOH bending, hydroxyl translations and water librations.

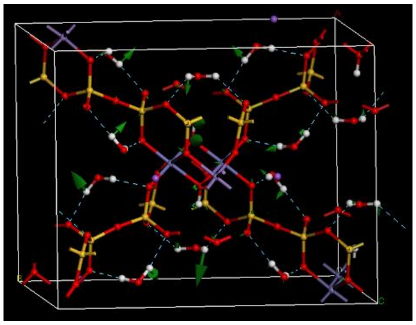

Table S5. Experimental and calculated infrared band wavenumbers of tuperssuatsiaite and calculated intensities and assignments.

Band Name	Exp. Freq. (cm^{-1})	Calc. Freq. (cm^{-1})	Int. $(D/Å)^2/amu$	Assignment
		**OH stretching region**		
a	3727	3684	261.5	ν(OH)
b	3625	3625	2066.1	"
c	3600	3604	559.0	"
d	3539	3506	4273.0	"
e	3497	3437	1733.8	"
		3433	1222.5	"
		3432	2384.4	"
		3431	2862.0	"
f	3458	3415	799.4	"
g	3437	3396	1073.4	"
h	3356	3371	785.3	"
i	3215	3250	1066.9	"
		**Water bending region**		
j	1650	1632	988.9	δ(HOH)
	1630	1620	306.5	"
	1615	1612	255.9	"
	1595	1607	544.1	"
		**390-1500 cm^{-1} region**		
α	1180	1153	739.3	ν(SiO)
β	1114	1105	907.2	ν(SiO) + δ(FeOH)
	1090	1094	2280.8	"
		1078	2717.6	ν(SiO)
γ	1031	1039	1742.6	"
δ	982	992	9183.3	δ(FeOH)
		968	7711.2	ν(SiO) + δ(FeOH)
		956	5752.2	δ(FeOH)
ε	948	934	25512.1	ν(SiO) + δ(FeOH)
		925	53693.4	δ(FeOH)
		918	13318.5	"
η	904	898	18608.8	"
θ	862	860	17546.1	ν(SiO) + δ(FeOH)
		856	8112.7	δ(FeOH)
λ	817	824	55464.8	ν(SiO) + δ(FeOH)
μ	775	803	4987.7	ν(SiO) + δ(FeOH) + $l(H_2O)$
		799	6357.9	ν(SiO) + $l(H_2O)$
ξ	764	742	6822.5	$l(H_2O)$
π	680	688	3253.3	ν(SiO) + ρ(SiOSi) + δ(FeOH) + $l(H_2O)$
	673	684	2581.3	ν(SiO) + ρ(SiOSi) + $l(H_2O)$
σ	635	645	2195.6	ν(SiO) + ω(OSiO) + $l(H_2O)$
	609	603	2291.8	ν(SiO) + ω(OSiO) + ρ(SiOSi) + δ(FeOH) + $l(H_2O)$
τ	567	541	3630.0	$l(H_2O)$
		523	3034.6	"
φ	520	515	8583.3	δ(FeOSi) + δ(SiOSi) + $l(H_2O)$
ϕ	431	437	7994.9	ν(FeO) + δ(NaOH) + T(OH) + $l(H_2O)$
	418	426	9807.3	$l(H_2O)$
ψ	395	408	6276.9	δ(NaOH) + $l(H_2O)$
		398	15023.3	ν(MnO) + δ(FeOH) + T(OH) + $l(H_2O)$

Table S6. Computed elastic constants of tuperssuatsiaite. All the values are given in GPa. The indices of the matrix elements of the matrix representation of the elasticity tensor are expressed using the standard Voigt notation. In this notation, a pair of Cartesian indices are contracted into a single integer ($1 \leq i \leq 6: xx \rightarrow 1, yy \rightarrow 2, zz \rightarrow 3, yz \rightarrow 4, xz \rightarrow 5, xy \rightarrow 6$).

ij	C_{ij}
11	37.48
22	181.25
33	178.99
44	46.12
55	28.10
66	38.19
12	37.53
13	24.04
14	-67.69
15	-4.80
16	-27.13
23	85.17
24	-79.38
25	-2.31
26	-28.46
34	-40.81
35	-0.95
36	-12.70
45	-2.11
46	-10.87
56	1.27

Table S7. Computed mechanical properties of tuperssuatsiaite (Voigt approximation). The values of the bulk, shear and Young moduli (B, G and E) are given in in GPa.

	**Property**	**Value**
$\boldsymbol{B}$	Bulk modulus	76.80
$\boldsymbol{G}$	Shear modulus	39.21
$\boldsymbol{E}$	Young modulus	100.53
$\boldsymbol{\nu}$	Poisson ratio	0.28
$\boldsymbol{D}$	Ductility index	1.95
$\boldsymbol{H}$	Hardness index	4.79
$\boldsymbol{A^U}$	Universal anisotropy	3.16

Table S8. Calculated compressibility, $k_V^{\nu_{min}} = -1/V \cdot (\partial V/\partial P_{\nu_{min}})_P$ of tuperssuatsiaite as a function of the external pressure applied along the direction of minimum Poisson's ratio.

**P (GPa)**	$\boldsymbol{k_V^{\nu_{min}}}$ **(TPa^{-1})**	**P (GPa)**	$\boldsymbol{k_V^{\nu_{min}}}$ **(TPa^{-1})**
-0.030	10.437	-0.010	-37.632
-0.025	3.948	-0.005	-38.131
-0.020	-9.324	0.000	-16.346
-0.015	-25.257	0.005	84.839

Table S9. Calculated compressibilities, $k_b = -1/b \cdot (\partial b/\partial P)_P$ and $k_c = -1/c \cdot (\partial c/\partial P)_P$, of tuperssuatsiaite as a function of the external isotropic pressure.

**P (GPa)**	$\boldsymbol{k_b}$**(TPa^{-1})**	$\boldsymbol{k_c}$**(TPa^{-1})**	**P (GPa)**	$\boldsymbol{k_b}$**(TPa^{-1})**	$\boldsymbol{k_c}$**(TPa^{-1})**
0.80	3.020	3.546	2.50	-5.345	1.137
0.90	2.363	3.584	2.60	-2.609	3.004
1.00	1.320	3.798	2.70	-0.476	3.994
1.10	0.236	4.103	2.80	1.556	3.858
1.20	-0.664	4.411	2.90	3.987	2.347
1.30	-1.275	4.635	3.00	7.319	-0.789
1.40	-1.613	4.689	3.10	12.060	-5.794
1.50	-1.815	4.485	3.20	10.284	-6.340
1.60	-2.136	3.936	3.30	5.304	-3.473
1.70	-2.952	2.953	3.40	1.747	-1.150
1.80	-4.758	1.449	3.50	-0.581	0.629
1.90	-8.168	-0.662	3.60	-1.879	1.866
2.00	-13.906	-3.466	3.70	-2.347	2.561
2.10	-22.804	-7.048	3.80	-2.185	2.714
2.20	-22.236	-7.260	3.90	-1.596	-
2.30	-14.645	-4.240	4.00	-0.778	-
2.40	-9.188	-1.360	4.10	0.067	-

Table S10. Position of the most intense reflections in the powder X-ray diffraction pattern of tuperssuatsiaite with CuK_α radiation (λ = 1.540598 Å): (A) Powder X-ray pattern determined from the computed unit cell; (B) Experimental powder X-ray diffraction pattern.

$[hkl]$	(A) Theoretical 2θ (°)	(A) Theoretical d (Å)	(B) Experimental 2θ (°)	$\Delta[2\theta]$ (°)
[1 1 0]	8.4120	10.5028	8.1946	0.22
[4 0 0]	27.6720	3.2211	26.2920	1.38
[-3 -5 1]	34.0080	2.6341	34.0734	-0.07
[2 2 1]	26.8710	3.3152	26.3129	0.56
[-2 6 1]	35.3840	2.5347	35.7700	-0.39
[2 4 0]	24.0210	3.7017	23.8308	0.19
[0 2 1]	19.9340	4.4506	19.8901	0.04
[-2 2 1]	21.4020	4.1484	21.5582	-0.16
[1 1 2]	38.0000	2.3660	37.5190	0.48
[1 3 1]	25.1740	3.5347	24.7419	0.43
[5 1 0]	35.1540	2.5508	33.2565	1.90
[0 2 0]	9.7630	9.0526	9.9017	-0.14
[0 4 0]	19.5970	4.5263	19.8424	-0.25
[-2 4 1]	27.4300	3.2490	27.4440	-0.01
[2 2 0]	16.8700	5.2514	16.2588	0.61
[-4 -6 1]	41.4800	2.1752	41.3835	0.10
[-1 5 1]	29.9210	2.9839	30.3450	-0.42
[0 0 2]	35.0790	2.5560	35.1102	-0.03
[3 5 1]	39.5900	2.2746	38.7758	0.81
[-7 -5 1]	54.4160	1.6847	52.9038	1.51
[-4 -2 1]	30.1440	2.9623	29.6014	0.54

Table S11. Main structural changes in tuperssuatsiaite under isotropic pressure. The meaning of the width and height (w_{ch} and h_{ch}) of a channel is illustrated in the subfigure (A) of Fig. 8. Distances, volumes and angles are given in Å, Å^3 and degrees, respectively.

**P (GPa)**	**0.894**	**1.490**	**2.032**	**2.509**
**R(Na − O)**				
Na − O10	2.253	2.230	2.218	2.204
Na − O2	2.264	2.301	2.287	2.249
Na − Ow5	2.226	2.256	2.256	2.221
Na − Ow1	2.246	2.481	2.463	2.410
Na − O1	2.505	2.521	2.507	2.400
Na − O9	2.535	2.598	2.594	2.542
$<$ Na − O $>$	2.338	2.398	2.387	2.338
**α(O − Na − O)**				
O10 − Na − O2	151.808	142.843	142.755	143.960
O10 − Na − Ow5	112.493	103.570	103.034	103.720
O10 − Na − Ow1	82.190	69.074	69.510	69.959
O10 − Na − O1	69.829	66.706	66.624	68.072
O10 − Na − O9	86.994	79.382	79.097	80.886
Ow5 − Na − Ow1	101.720	93.590	95.940	91.395
Ow1 − Na − O1	97.366	106.352	106.587	105.830
O1 − Na − O9	87.147	67.513	68.011	73.720
O9 − Na − Ow5	84.125	87.604	83.354	82.752
**Channel dimensions**				
w_{ch}	8.223	7.764	7.858	8.350
h_{ch}	6.561	5.637	5.413	4.720
**Lattice parameters**				
a	13.2685	12.3125	12.1208	11.4369
b	18.0707	18.0733	18.1281	18.2917
c	5.2984	5.2850	5.2809	5.2940
Vol.	1221.9305	1118.3967	1100.8185	1039.9044

References

1. Cámara, F., Garvie, L. A. J., Devouard, B., Groy, T., Busec, P. R. The structure of Mn-rich tuperssuatsiaite: A palygorskite-related mineral. *Am. Mineral*. **87**, 1458–1463, https://doi.org/10.2138/am-2002-1023 (2002).
